# Diabetic kidney disease in children and adolescents: an update

**DOI:** 10.1007/s00467-021-05347-7

**Published:** 2021-12-16

**Authors:** Lauren N. Lopez, Weijie Wang, Lindsey Loomba, Maryam Afkarian, Lavjay Butani

**Affiliations:** 1 Division of Nephrology, Department of Internal Medicine, University of California, Davis, Sacramento, CA USA; 2 University of California, Berkeley, Berkeley, CA USA; 3 Division of Pediatric Endocrinology, Department of Pediatrics, University of California, Davis, Sacramento, CA USA; 4 Division of Pediatric Nephrology, Department of Pediatrics, University of California, Davis, 2516 Stockton Blvd, Room 348, Sacramento, CA 95817 USA

**Keywords:** Diabetes, Nephropathy, Microalbuminuria, Metabolic syndrome

## Abstract

Diabetic kidney disease (DKD), previously encountered predominantly in adult patients, is rapidly gaining center stage as a childhood morbidity and one that pediatric nephrologists are likely to encounter with increasing frequency. This is in large part due to the obesity epidemic and the consequent rise in type 2 diabetes in children and adolescents, as well as the more aggressive diabetes phenotype in today’s youth with more rapid β-cell decline and faster development and progression of diabetes-related complications along with lower responsiveness to the treatments used in adults. DKD, an end-organ complication of diabetes, is at the very least a marker of, and more likely a predisposing factor for, the development of adverse cardiovascular outcomes and premature mortality in children with diabetes. On an optimistic note, several new therapeutic approaches are now available for the management of diabetes in adults, such as GLP1 receptor agonists, SGLT2 inhibitors, and DPP4 inhibitors, that have also been shown to have a favorable impact on cardiorenal outcomes. Also promising is the success of very low-energy diets in inducing remission of diabetes in adults. However, the addition of these pharmacological and dietary approaches to the management toolbox of diabetes and DKD in children and adolescents awaits thorough assessment of their safety and efficacy in this population. This review outlines the scope of diabetes and DKD, and new developments that may favorably impact the management of children and young adults with diabetes and DKD.

## Introduction

The prevalence of diabetes, and its antecedent insulin-resistant phenotype, continues to rise in children and adolescents, with an annual percentage change of 1.9% per year for type 1 diabetes (T1D), and 4.8% per year for type 2 diabetes (T2D) from 2002 to 2015; this rise is even more pronounced among racial and ethnic minorities [1, 2] and, for T2D, parallels the increase in the prevalence of obesity. In addition, compared to adults, diabetes has a more aggressive clinical course in children and adolescents, marked by a muted response to current interventions [3, 4], as well as faster loss of β-cell function, progression of insulin resistance, and development of end-organ complications [5, 6]. As a consequence, the prevalence of diabetic kidney disease (DKD) is increasing in children and adolescents, rising from 1.16 to 3.44% between 2002 and 2013 [7], particularly in youth with T2D who, compared to age-matched controls, have been noted to have a 23-fold increased risk of kidney failure and a 39-fold increased risk of dialysis [8]. Of equal concern is the observation that DKD marks a subset of people at especially high risk of cardiovascular disease and premature mortality [9, 10]. As such, diabetes and its complications, particularly DKD, continue to gain more importance for pediatric nephrologists. Here, we review recent updates to the diagnosis and management of DKD in children and adolescents.

## Diagnosis

### Current clinical markers

As with many other chronic kidney diseases (CKD), the diagnosis of DKD hinges on changes in urinary albumin excretion rate (AER) and glomerular filtration rate (GFR). Structural changes characteristic of DKD are observed in kidney biopsies as early as the first few years after the onset of diabetes [11], but the disease remains clinically silent for at least the first 10–15 years.

#### Hyperfiltration

Defined as a GFR of 120–150 mL/min/1.73m^2^ or > 2 standard deviations above the mean normal GFR [12], hyperfiltration is reported in 25–40% of youth with diabetes [6]. Hyperfiltration was initially attributed to early increases in kidney plasma flow and intraglomerular pressure and is believed to be a strong predictor of subsequent GFR loss and DKD progression [13]. However, the evidence supporting the association between hyperfiltration and albuminuria has been called into question more recently, in both T1D and T2D, having been observed in some studies (T1D, T2D [14]) but not in others (T1D [15], T2D [16]). Hyperfiltration has been associated with GFR loss in both T1D [17] and T2D [14], but only one study documented a decline to a GFR < 60 mL/min/1.73m^2^ [18], leaving open the possibility that the observed rapid GFR drop was either reversal of the early glomerular hyperfiltration or regression to the mean. Importantly, early hyperfiltration was not associated with a greater risk of subsequent progression to an estimated GFR of < 60 mL/min/1.73m^2^ in people with T1D in the seminal Diabetes Control and Complications Trial/Epidemiology of Diabetes Interventions and Complications (DCCT/EDIC) study [19]. As the authors pointed out, this observation does not counter the experimental data showing a deleterious effect of single-nephron hyperfiltration and the resulting glomerular hypertension. As discussed later, current recommendations do not support angiotensin blockade purely for the management of hyperfiltration.

#### Albuminuria

Increases in urinary AER above normal were classically described as the earliest sign of DKD. Microalbuminuria, defined as a urinary AER 30–299 mg/day (or urine albumin-to-creatinine ratio 30–299 mg/g in spot urine specimens), occurs in 26% and 51% of children and adolescents after 10 and 19 years of diabetes, respectively [20]. Macroalbuminuria, defined as a urinary AER ≥ 300 mg/day (or urine albumin-to-creatinine ratio ≥ 300 mg/g in spot urine specimens), was reported in 14% of children with T1D after a median diabetes duration of 10 years [20]. Evidence suggests that albuminuria occurs earlier and progresses more rapidly in children and adolescents with T2D [21]. However, use of micro- or macroalbuminuria as surrogate markers for DKD has been challenged. The DCCT/EDIC participants with microalbuminuria had a much higher chance of regression to normoalbuminuria (10-year cumulative incidence of 40%) than of progression to macroalbuminuria (28%), CKD stage 3 or higher (15%), or kidney failure (4%) [22]. Macroalbuminuria was associated with a greater risk of DKD progression, but even this higher level of albuminuria led more often to normoalbuminuria (48% 10-year cumulative risk), than of progression to CKD stage 3 or higher (32%) or kidney failure (16%) [23]. In addition, while most cases of GFR loss are preceded by albuminuria, in a sizable minority (24%), GFR loss occurs in the absence of albuminuria [24], further challenging the utility of albuminuria as a key predictor of DKD progression. Despite these caveats, pediatric guidelines recommend annual screening for detection of albuminuria, using a urine sample collected in the morning, starting at puberty or 10 years of age (whichever is earlier) beginning 5 years after T1D diagnosis, and upon T2D diagnosis [25, 26] (see Table 1).

**Table 1:** Guidelines for screening and key interventions in children and adolescents with diabetes

	Type 1 diabetes	Type 2 diabetes	Caveats
HbA1c target	< 7.5%	< 7%	Individualized target based on risk–benefit of intervention and patient-family factors
BP target	< 90^th^ percentile (screen at each visit)		Pharmacologic agent of choice – ACEi or ARB
Urine microalbumin/creatinine ratio	Start at puberty or 10 yrs of age (whichever is earlier) once the child has had diabetes for 5 yrs (Screen annually)	Start at diagnosis (Screen annually)	Treat with ACEi/ARB if two of three MORNING urines > 30 mg/g, over 6 months, after efforts to improve glycemic control and achieve target BP
eGFR	Screen at diagnosis and annually		

*ACEi*, angiotensin-converting enzyme inhibitor; *ARB*, angiotensin receptor blocker; *BP*, blood pressure; *eGFR*, estimated glomerular filtration rate

#### GFR loss

Also recommended is monitoring GFR, calculated from serum creatinine using estimating equations, at baseline and intermittently thereafter, based on clinical status, age, and the duration and treatment of diabetes [27]. All existing GFR estimating equations are imperfect, particularly in children and adolescents with T1D and those with normal or higher than normal GFRs [28], necessitating serial eGFR determinations using a consistent, age-appropriate and validated eGFR calculator; for youth > 18 years of age, an adult eGFR calculator should be utilized. In children with GFR < 60 mL/min/1.73m^2^, a 4-variable kidney failure risk equation (including age, sex, AER, and eGFR) was able to predict 1–5-year kidney failure risk, supporting the importance of eGFR and albuminuria as the predominant determinants of kidney failure risk in children with advanced CKD [29]. However, this level of CKD has thus far been less commonly seen in pediatric diabetes, though its prevalence may be rising with the increase in T2D.

In summary, our current understanding of the trajectory of DKD in children and adolescents suggests that advanced CKD and kidney failure take decades to develop after the onset/diagnosis of diabetes, which means that the data on the prevalence and time course of these outcomes in childhood-onset diabetes is largely derived from adult studies [30]. This presents a dilemma for any rigorous study of DKD in children and adolescents because understanding any aspect of DKD (biomarkers, risk factors for progression, response to interventions, etc.) has had to rely on intermediate outcomes (e.g., albuminuria, hyperfiltration) [30], which are not reliable surrogates for DKD progression, or else on extrapolation from studies in adult patients with diabetes.

### Novel biomarkers

The lack of reliable surrogate markers for DKD progression during childhood and adolescence makes identification of novel markers of early disease in youth even more critical than it is in adults. Most published studies report cross-sectional associations between various urinary/serum protein biomarkers and intermediate outcomes such as albuminuria, with a smaller number of studies examining these associations using longitudinal data [31–33]. Rare studies are notable in bypassing the reliance on these flawed surrogate markers and examining the association between putative biomarkers such as plasma advanced glycation end-products [34] or plasma bradykinin [35] with early kidney structural changes in youth with T1D. In adults, serum tumor necrosis factor receptor 1 (TNFR1) and TNFR2 have been found to be associated with early structural changes of DKD as well as with DKD progression [36], highlighting the contribution of inflammatory pathways to the disease process. Other potential biomarkers for DKD, based on adult studies, are urinary neutrophil gelatinase–associated lipocalin (NGAL), kidney injury molecule-1 (KIM-1), N-acetyl-β-d-glucosaminidase (NAG), and liver fatty acid–binding protein (LFABP). Sometimes referred to as “kidney tubular injury markers,” these urinary proteins have been associated with DKD progression in some [37] but not in other [38] studies. None of these putative markers is currently a part of routine clinical care in adult or pediatric DKD.

### Monogenic forms of diabetes

While a comprehensive discussion of this subject [39] is beyond the scope of our manuscript, it is worth mentioning that monogenic forms of diabetes, which represent an uncommon group of single-gene disorders characterized by functional defects of pancreatic β-cells, are being increasingly recognized as a result of advancements in genomic medicine. Monogenic forms of diabetes include neonatal diabetes mellitus, maturity-onset diabetes of the young, and mitochondrial diabetes, to name a few. Mutations in over 30 different genes are associated with monogenic diabetes; some of these have concomitant kidney anomalies. Awareness of monogenic forms of diabetes among pediatricians is important because of their screening and therapeutic implications, allowing for personalized medicine.

### Diabetic ketoacidosis-associated acute kidney injury

A more recently noted aspect of the DKD trajectory is the recurrent episodes of acute kidney injury (AKI) and their contribution to DKD progression. In particular, AKI is a common consequence of diabetic ketoacidosis (DKA), occurring in 43–64% of pediatric patients with DKA [40]. Risk factors for DKA-associated AKI include older age, higher heart rate, higher blood urea nitrogen (BUN), higher glucose-corrected serum sodium concentration, higher glucose concentration, and lower pH, all on initial presentation [41]. In addition, a prior episode of DKA-associated AKI substantially increases the risk of subsequent similar episodes, suggesting that either children vary in their risk for AKI or that previous episodes of AKI increase kidney susceptibility to subsequent injury [41]. DKA frequently predisposes to AKI by volume depletion and renal hypoperfusion due to osmotic diuresis and occasionally gastrointestinal losses, contributing to a pre-renal picture. However, the greater severity of AKI in some children (stage 2 or 3) suggests the possibility that kidney injury has progressed from merely pre-renal to acute tubular injury [42].

In adults with diabetes, AKI is associated with a greater risk of developing DKD, and this risk increases with each AKI episode [43]. Compared to adults with DKA who do not experience AKI, those who do have a more rapid progression of DKD as well as a higher long-term mortality [44]. Furthermore, the rate of CKD progression is proportionate to the severity of the DKA-associated AKI [44]. Mechanisms driving the transition from AKI to CKD may include the development of tubulointerstitial fibrosis following proximal tubular injury, glomerular endothelial dysfunction, oxidative stress, ongoing inflammation, and cell cycle arrest [45]. In children, data regarding the association between DKA-associated AKI and DKD progression are currently lacking.

## Management

### Addressing risk factors that promote the development of DKD

The seminal DCCT/EDIC study highlighted *hyperglycemia* as the primary causal mechanism for diabetes-related complications in children and adolescents based on the reduction of microvascular complications by intensive diabetes treatment in 195 DCCT participants, who were 13–17 years old, similar to what was noted in the entire cohort [46]. Based on this study, the HbA1c target has been set at < 7.0–7.5% for patients with T1D and T2D by national and international societies [25, 26]. However, in determining glycemic targets for diabetes treatment, one consideration is worth noting. Several large observational studies have found that the glycemic threshold with the lowest risk of end-organ complications was at an HbA1c nadir of 5.0–5.5% [47, 48]. However, while lowering HbA1c to 7% led to a reduction of complications [49], no interventional trial has shown improvement in clinical outcomes by lowering HbA1c below 7 (e.g., VADT [50], ADVANCE [51]) and some have shown harm (ACCORD [52]). An explanation for this apparent dichotomy between observational and interventional studies may be that the optimal glycemic target is determined by a balance of the benefits vs. risks for any given intervention. While a naturally occurring HbA1c of 5.0–5.5% may be associated with the lowest risk of complications, when HbA1c is pushed from 7 to 5.5% using more intensive diabetes treatments, these higher risks (e.g., hypoglycemia, weight gain) may negate any clinical benefits from the lower HbA1c threshold. On the other hand, with the use of treatments that have lower risks such as hypoglycemia (e.g., metformin or SGLT2 inhibitors) targeting a lower HbA1c threshold may be desirable to achieve improved outcomes. As such, the optimal glycemic target is likely not a fixed threshold, but one that varies based on the balance of the risk vs. benefit of each specific intervention used to achieve that glycemic threshold. Table 1 outlines recommended guidelines in the management of children and adolescents with T1D and T2D.

Previously a feature of T2D, but becoming more prevalent in both T1D and T2D, *insulin resistance* (IR) has been strongly linked to the development of DKD. IR can initiate kidney injury independently of [53], and even prior to progression to [54] frank hyperglycemia. IR is well-known to be augmented by the growth and hormonal changes that occur during puberty. For example, IR is prominent in adolescents with T1D even in the absence of adiposity [55]. The rise of obesity in youth has further added to the puberty-induced increase in IR resulting in rapid progression of IR to diabetes in this population, as shown in the TODAY [56] and RISE [57] studies.

*Hypertension*, a well-established risk factor for the development of DKD [58], increases in incidence rapidly in youth with diabetes, particularly T2D [56]. This rise is unaffected by glycemic control and requires multiple medications in a sizable fraction of patients, suggesting that obesity and diabetes contribute significantly to treatment-resistant hypertension [56]. Current guidelines [59] recommend that pharmacotherapy be instituted promptly, along with lifestyle measures, for treatment of hypertension (BP > 95^th^ percentile). Recommendations are also that first-line agents in people with diabetes are angiotensin-converting enzyme inhibitors (ACEis) or angiotensin receptor blockers (ARBs), following the provision of reproductive counseling to women of childbearing age. The target BP in all patients should be less than the 90^th^ percentile. However, in general, hypertension often goes underdiagnosed in children, making this a critical target for research and innovations to improve recognition of this important risk factor for DKD.

*Serum uric acid (SUA)* has long been considered a likely causal risk factor for DKD in both adults and adolescents [60]. Furthermore, SUA reduction in small clinical trials was associated with a slower decline in GFR in patients with CKD [61], raising hopes that targeting SUA may yield a novel strategy for slowing DKD progression. Unfortunately, two recent clinical trials targeting SUA reduction with allopurinol (PERL [62]) and febuxostat (FEATHER [63]) showed no impact on DKD/CKD progression.

### Dietary and lifestyle interventions

Current standards of care include dietary education, e.g., carbohydrate counting, calorie tracking and consumption of low glycemic index foods [25], and promotion of increased physical activity, all of which are associated with improved glycemic control [64]. However, these strategies continue to have limited success. For example, only 20% and 30% of adolescents with T2D limit high-fat foods and use carbohydrate counting, respectively [64]. Dietary recommendations in youth must also consider the high prevalence of disordered eating behavior in youth with diabetes [65], itself associated with worse glycemic control and greater adverse outcomes. The American Diabetes Association (ADA) guidelines also recommend weight control, particularly for youth with T2D [25]. However, intensive lifestyle intervention programs that combine diet and exercise goals have thus far been unsuccessful in promoting significant weight loss or improvement in glycemic control in adolescents with T2D [66].

More recently, the notable success of DiRECT (Diabetes REmission Clinical Trial) has re-ignited interest in the dietary approach to diabetes management, particularly with use of very low-energy diets (VLED). Obese adults with recent-onset T2D, fed a very low calorie (825–835 kcal/day) meal replacement diet for 3–5 months, followed by structured re-introduction of food and monthly support, achieved an astounding 46% diabetes remission rate at 12 months, with a decline in remission rate to 36% at 24 months [67]. While these results have generated significant excitement as well as a recommendation for VLED utilization in some patient populations, their safety and efficacy in adolescents have not been well-studied to date. A recent systematic review and meta-analysis of VLEDs in children and adolescents demonstrated weight loss and improved cardio-metabolic outcomes, with several caveats [68]: VLED safety could not be assessed because adverse events were inadequately described in most of the analyzed studies. Furthermore, of the 24 identified studies, only four were published since the year 2000, only two were randomized controlled trials, and only two focused on patients with a diagnosis of T2D [68]. As such, despite their potential long-term benefits, inclusion of VLEDs in the current management of diabetes in children and adolescents awaits support from larger and controlled trials and cannot be recommended at this time due to the potential for adverse effects in this highly vulnerable population where growth and neurodevelopmental considerations are of utmost importance.

### Pharmacological management

There have been significant advances in pharmacologic treatments for adults with diabetes over the past few decades [69, 70]. Several glucose-lowering medications from three novel classes have been approved in the USA for use in adult patients with diabetes: glucagon-like peptide-1 (GLP1) receptor agonists, dipeptidyl peptidase-4 (DPP4) inhibitors, and sodium glucose cotransporter-2 (SGLT2) inhibitors [71], many of which have beneficial effects on kidney outcomes [69]. However, children and adolescents with diabetes are yet to benefit from these advances with primary diabetes treatments in children being limited to metformin and insulin [72]. While metformin is an effective glucose-lowering agent without the risk of hypoglycemia, it does not substantially preserve β-cell function such that over time glycemic function tends to worsen in patients. In spite of early fears related to the association between metformin and lactic acidosis in patients with CKD, data to support this complication have been inconsistent such that the FDA revised its warning regarding metformin use in patients with CKD who have an eGFR > 45 mL/min per 1.73 m^2^; dose adjustments are required with a decline in eGFR and there are currently no safety data for metformin use in patients with an eGFR < 30 mL/min per 1.73 m^2^ or in those on dialysis [73].

To date, only one novel drug, a GLP1 receptor agonist, has been approved for children and adolescents. Given the rising prevalence of diabetes as well as DKD [7, 8] in children and adolescents, the increase in lifetime adverse outcomes and premature mortality in this population, and the salutary effects of some of the novel diabetes medications on adverse cardiorenal outcomes in adults, it becomes critical to prioritize studies needed to furnish data on the safety and efficacy of these agents in children and adolescents. The current limited and small pediatric trials of these agents have been conducted only pursuant to the *pediatric rule*, an FDA mandate that all medications approved for use in adults must also undergo safety and efficacy testing in pediatric patients. As such, pediatric participant numbers are miniscule (≤ 300) (Tables 2 and 3), in stark contrast to the respective adult trials that have enrolled thousands of participants. Furthermore, few results from these pediatric trials have been published in peer-reviewed journals.

**Table 2:** Clinical trials of GLP1 receptor agonists, DPP4 inhibitors, and SGLT2 inhibitors in children and adolescents with type 1 diabetes. List of all ongoing (in *italics*) and completed trials of GLP1 receptor agonists, DPP4 inhibitors, and SGLT2 inhibitors in children and adolescents < 18 years in the USA. Participant numbers listed in parentheses indicate target enrollment numbers for ongoing trials

Drug class	Major side effects	Experimental drug	Design	Ph	*N*	Duration	Primary and secondary outcomes	Mortality and SAE	Results
GLP1 receptor agonists	Nausea, vomiting and diarrhea Injection site reactions	Exenatide NCT01269034	Randomized Open label	4	14	1d per treatment (× 3)	1) Postprandial hyperglycemia 2) Postprandial glucagon and gastric emptying	Not available	Completed: 2017 Results: not published
		NCT01269047	Randomized Open label	4	37	16 wk	1) Postprandial blood glucose 2) Difference in HbA1c between control and treatment groups	Mortality: none SAE: none	Completed: 2016 Results: NCT01269047
		NCT00456300	Randomized Double blind	2	9	1d per treatment (× 3)	1) Mean postprandial plasma glucose AUC	Mortality: none SAE: none	Completed: 2009 Results: [74]
		Liraglutide *NCT02516657*	*Non-randomized* *Open label*	*3*	*(15)*	*2 wk*	*1) Mean weekly blood glucose* *2) TDID, blood glucose, amylase*	*Ongoing study*	*Estimated completion: 2021*
DPP4 inhibitors	Upper respiratory tract infections Urinary tract infections Arthralgias	Sitagliptin NCT01718093	Randomized Open label	4	21	6 wk	1) Glucose concentration and AUC	Not available	Completed: 2015 Results: not published
		NCT01155284	Randomized Triple blind	2	68	12 mo	1) 2 h C-peptide AUC at month 12 2) 2 h C-peptide AUC at month 6	Mortality: none SAE: sitagliptin 2/46, placebo 1/22	Completed: 2015 Results: [75]
SGLT2 inhibitors	Normoglycemic ketoacidosis Volume depletion Genital infections	Dapagliflozin *NCT04333823*	*Randomized* *quadruple blind*	*3*	*(100)*	*16 wk*	*1) Change in mGFR from baseline to wk 16* *2) HbA1c, AE, glycemic control, weight, TDID*	*Ongoing study*	*Estimated completion: 2023*

*AE*, adverse events; *AUC*, area under the curve; *d*, days; *h*, hours; *mGFR*, measured glomerular filtration rate; *mo*, months; *Ph*, phase; *SAE*, serious adverse events; *TDID*, total daily insulin dose; *wk*, weeks

**Table 3:** Clinical trials of GLP1 receptor agonists, DPP4 inhibitors, and SGLT2 inhibitors in children and adolescents with type 2 diabetes. List of all ongoing (in *italics*) and completed trials of GLP1 receptor agonists, DPP4 inhibitors, and SGLT2 inhibitors in children and adolescents < 18 years in the USA. Participant numbers listed in parentheses indicate target enrollment numbers for ongoing trials

Drug class	Experimental drug	Design	Ph	*N*	Duration	Primary and secondary outcomes	Mortality and SAE	Results
GLP1RA	Liraglutide *NCT02960659*	*Randomized* *Open label*	*1*	*(102)*	*12 wk*	*1) Change in stool short chain fatty acids, change in gluconeogenesis* *2) Change in glucose production, change in gut microbiota diversity*	*Ongoing study*	*Estimated completion: 2022*
	NCT01541215	Randomized Double blind	3	135	26 wk + 26 wk open-label extension	1) Change in HbA1c from baseline to wk 26 2) Various related to glycemic control, biochemistry, development, AE	Mortality: none SAE: placebo 4/68, liraglutide during treatment 9/66, liraglutide during f/u 7/52	Completed: 2020 Results: [76]
	NCT00943501	Randomized Double blind	1	21	5 wk + 3 wk AE f/u	1) Number and type of AE 2) PK, PD	Mortality: none SAEs: none	Completed: 2011 Results: [77, 78]
	Dulaglutide *NCT02963766*	*Randomized* *Double blind*	*3*	*154*	*60 wk*	*1) Change in HbA1c from baseline to wk 26* *2) Various related to glycemic control, AE, PK*	*Ongoing study*	*Estimated completion: 2022*
	Exenatide *NCT01554618*	*Randomized* *Quadruple blind*	*3*	*84*	*24 wk* + *28 wk open-label extension*	*1) Change in HbA1c from baseline to wk 24, AE, ADA* *2) Various related to glycemic control, biochemistry, AE, PK*	*Ongoing study*	*Estimated completion: 2023*
	NCT00658021	Randomized Double blind	3	122	28 wk	1) Change in HbA1c from baseline to wk 28, AESI 2) Various related to glycemic control, biochemistry	Mortality: none SAE: 5 μg 3/41, 10 μg 1/37, placebo 1/42	Completed: 2020 Results: not published
	NCT00950677	Randomized Single blind	4	16	4 h	1) Glucose concentration and AUC 2) Glucagon, gastric emptying, PK	Not available	Completed: 2011 Results: not published
	NCT00254254	Randomized Single blind	2	13	1d per treatment (× 3)	1) PK, PD, AE	Mortality: none SAE: 1/13	Completed: 2007 Results: [79]
	Lixisenatide NCT02803918	Randomized Double blind	1	23	6 wk + 4 wk f/u	1) AE, TEAE, ADA 2) PK, PD	Not available	Completed: 2020 Results: not published
	NCT01572649	Randomized Double blind	1	24^ǂ^	1d per treatment (× 3)	1) Plasma glucose AUC 2) PK, PD	Not available	Completed: 2014 Results: not published
	Semaglutide *NCT04596631*	*Randomized* *Quadruple blind*	*3*	*(132)*	*52 wk*	*1) Change in HbA1c baseline to wk 26* *2) Various related to glycemic control, weight, metabolic, biochemistry, development*	*Ongoing study*	*Estimated completion: 2025*
DPP4i	Alogliptin *NCT02856113*	*Randomized* *Quadruple blind*	*3*	*(150)*	*52 wk*	*1) Change in HbA1c from baseline to wk 26* *2) Various related to safety*	*Ongoing study*	*Estimated completion: 2021*
	NCT00957268	Non-randomized Open label	1	24^†^	72 h	1) PK 2) PK, PD	Mortality: none SAE: none	Completed: 2013 Results: not published
	Linagliptin *NCT03429543*	*Randomized* *Double blind*	*3*	*(186)*	*26 wk* + *26 wk safety extension*	*1) Change in HbA1c baseline to wk 16, treatment failure at wk 26* *2) Various related to glycemic control, metabolic*	*Ongoing study*	*Estimated completion: 2023*
	NCT01342484	Randomized Double blind	2	40	12 wk	1) Change in HbA1c from baseline to wk 12 2) % DPP4 inhibition, FPG	Mortality: none SAE: placebo 1/15, linagliptin 1 mg 0/10, linagliptin 5 mg 0/14	Completed: 2016 Results: [80]
	Saxagliptin *NCT03199053*	*Randomized* *Double blind*	*3*	*(254)*	*52 wk*	*1) Change in HbA1c from baseline to wk 26* *2) FPG, HbA1c*	*Ongoing study*	*Estimated completion: 2023*
	NCT01204775	Randomized Double blind	3	8	52 wk	1) Change in HbA1c from baseline to wk 16	Mortality: none SAE: placebo 1/4, saxagliptin 0/4	Completed: 2016 Results: NCT01204775
	NCT01434186	Randomized Double blind	3	6	52 wk	1) Change in HbA1c from baseline to wk 16	Mortality: none SAE: none	Completed: 2016 Results: NCT01434186
	Sitagliptin NCT01485614	Randomized Double blind	3	200	20 wk placebo-controlled then 34 wk active-controlled	1) Change in HbA1c from baseline to wk 20, AE 2) Various related to glycemic control, metabolic, biochemistry, development	Mortality: 1 in sitagliptin group SAE: sitagliptin 10/95, placebo/metformin 7/90, metformin 1/9, placebo/sitagliptin 3/5	Completed: 2019 Results: NCT01485614
	NCT00730275	Randomized Double blind	1	35	72 h + 10–14d AE f/u	1) AE, sitagliptin AUC 2) PK, PD	Mortality: none SAE: none	Completed: 2019 Results: [81]
	Sitagliptin/metformin XR combined NCT01557504	Randomized Open label	1	25	9d + 14d AE f/u	1) Ability to swallow medication, PK, PD	Mortality: none SAE: none	Completed: 2014 Results: NCT01557504
SGLT2i	*Canagliflozin* *NCT03170518*	*Randomized* *Double blind*	*3*	*(146)*	*26 wk*	*1) Change in HbA1c from baseline to wk 26* *2) FPG, HbA1c, rescue therapy, body weight, biochemistry, development, UACR*	*Ongoing study*	*Estimated completion: 2023*
	NCT02000700	Non-randomized Open label	1	17	14d	1) Plasma concentration of drug after multiple doses 2) Plasma glucose, glucose excretion, tablet acceptability, AE	Not available	Completed: 2016 Results: not published
	Dapagliflozin *NCT03199053*	*Randomized* *Double blind*	*3*	*(254)*	*52 wk*	*1) Change in HbA1c from baseline to wk 26* *2) FPG, HbA1c*	*Ongoing study*	*Estimated completion: 2023*
	NCT02725593	Randomized Double blind	3	72	24 wk + 28 wk safety extension	1) Change in HbA1c from baseline to wk 24 2) FPG, rescue medication, HbA1c	Mortality: none SAE: 10 mg/10 mg 2/39, placebo/10 mg 3/33	Completed: 2020 Results: NCT02725593
	NCT01525238	Randomized Open label	1	24	48 h	1) PK 2) PK, various related to glycemic control, safety	Mortality: none SAE: none	Completed: 2014 Results: [82]
	Empagliflozin *NCT03429543*	*Randomized* *Double blind*	*3*	*(186)*	*26 wk* + *26 wk safety extension*	*1) Change in HbA1c baseline to wk 16, treatment failure at wk 26* *2) Various related to glycemic control, metabolic*	*Ongoing study*	*Estimated completion: 2023*
	NCT02121483	Randomized Open label	1	27	3d	1) PK 2) Urinary glucose excretion, FPG, plasma glucose profile	Mortality: none SAE: none	Completed: 2016 Results: [83, 84]
	Ertugliflozin *NCT04029480*	*Randomized* *Double blind*	*3*	*(150)*	*54 wk*	*1) Change in HbA1c from baseline to wk 24, AEs* *2) HbA1c, FPG*	*Ongoing study*	*Estimated completion: 2025*

Abbreviations: *ADA*, anti-drug antibodies; *AE*, adverse events; *AESI*, adverse events of special interest; *AUC*, area under the curve; *d*, days; *FPG*, fasting plasma glucose; *h*, hours; *PD*, pharmacodynamics; *PK*, pharmacokinetics; *Ph*, phase; *SAE*, serious adverse events; *TEAE*, treatment-emergent adverse events; *wk*, weeks

^ǂ^NCT01572649 enrolled 24 participants but not all were < 18y (at least 3 patients below 15 years and no more than 3 patients aged between 16 and 18 years)

^†^NCT00957268 enrolled 46 participants but only 24 were < 18y

#### GLP1 receptor agonists for T2D

GLP1 receptor agonists mimic the incretin system by stimulating glucose-dependent insulin secretion, inhibiting glucagon secretion and gastric emptying, and suppressing appetite, leading to a reduction in blood glucose levels [85] and some weight loss. The use of GLP1 receptor agonists is associated with a 17% reduction in risk of composite kidney outcomes including incident macroalbuminuria, reduced eGFR, progression to dialysis, or death from kidney causes, without increasing the incidence of hypoglycemia [85]. Six GLP1 receptor agonists have been approved in the USA for the treatment of T2D in adults in the past 5–10 years; only one (liraglutide) has been approved for use in patients age ≥ 10 years with T2D. A small 5-week dose escalation study in youth showed that liraglutide had a similar pharmacokinetic profile as that in adults with no serious adverse events in that short follow-up period [77]. It was also shown to improve glycemic control in youth with T2D, in conjunction with metformin [76], leading to FDA approval in June 2019. The Evaluation of Liraglutide in Pediatrics with Diabetes (ELLIPSE) trial in predominantly white, obese adolescents with T2D showed that the addition of liraglutide to metformin improved glycemic control [76]. A 3-month study examining liraglutide efficacy in 100 black youth and young adults with T2D (NCT02960659) is to be completed by the end of 2022. It is worth noting that FDA approval of liraglutide in children and adolescents is based on two trials involving a total of 156 participants, in contrast to adult trials with thousands of participants (e.g., *n* = 9,340 for liraglutide in LEADER).

#### GLP1 receptor agonists for T1D

No GLP1 receptor agonists are approved for use in children with T1D. The studies that have been completed thus far, and the one that is ongoing, are all small (< 40 participants) and have largely remained unpublished (Table 2).

#### DPP4 inhibitors for T2D

Like GLP1 receptor agonists, DPP4 inhibitors act on the incretin system. However, DPP4 inhibitors act to prevent the degradation of incretins by inhibiting the DPP4 enzyme [86]. This leads to an increase in postprandial GLP1 which stimulates insulin secretion, decreasing plasma glucose concentration. Unlike GLP1 receptor agonists, DPP4 inhibitors do not affect appetite or gastric emptying and are administered orally, rather than via the subcutaneous route [86]. DPP4 inhibitors provide effective glycemic control in patients with T2D but do not confer cardiovascular protection and potential benefits on kidney outcomes are controversial [87]. None has yet been approved for use in children or adolescents.

A dose-dependent reduction in mean HbA1c was noted in a small 12-week study of linagliptin, a DPP4 inhibitor, in 37 adolescents with T2D compared with placebo, but this difference was not statistically significant. There were no serious adverse events in the intervention group [80]. The ongoing DINAMO study (NCT03429543) is currently assessing the longer-term safety and efficacy of once daily linagliptin over 52 weeks and is estimated to be completed in 2023. The study aims to enroll 186 participants aged 10–17 years with T2D and BMI ≥ 85^th^ percentile for age and sex who will be randomized 1:1:1 to placebo, linagliptin, or empagliflozin (discussed in more detail below).

Similarly, trials with other DPP4 inhibitors have not shown statistically significant improvements in HbA1c. None of these trials has made it to publication, likely due to the very small population size. Additional larger trials are underway (Table 3).

#### DPP4 inhibitors for T1D

Even in adults, DPP4 inhibitors are currently only approved to treat T2D, while their efficacy in adults with T1D is being investigated [88]. In adolescents with T1D, the DPP4 inhibitor sitagliptin did not lead to significant improvement in 2-h c-peptide response following a meal challenge [75]. Another study (NCT01718093) in 21 adolescents investigating the glucose-lowering effects of adding sitagliptin, metformin, or both, to an insulin regimen, was completed in 2015 but results have not yet been reported.

#### SGLT2 inhibitors for T2D

SGLT2 inhibitors block sodium and glucose reuptake in the proximal tubules of the kidney, resulting in increased urinary sodium and glucose excretion leading to improvements in metabolic and hemodynamic parameters, including hyperglycemia, body weight, adiposity, and BP [89]. In addition, SGLT2 inhibitors have been shown to attenuate renal hyperfiltration in subjects with diabetes, by affecting tubular-glomerular feedback mechanisms. The cardiorenal benefits of SGLT2 inhibitors in adults have been well-established in dedicated cardiovascular outcome trials. There is also data showing SGLT2 inhibitors significantly lower the risk of kidney adverse outcomes, such as sustained decline in eGFR, need for dialysis, or death from kidney causes in adults [90, 91]. SGLT2 inhibitors are associated with an increased risk of genital infections [90, 91]. In the past decade, four SGLT2 inhibitors have been approved in the USA for adults with T2D and are highly recommended for patients with T2D and kidney disease to slow progression to dialysis [71]; however, no SGLT2 inhibitor has been approved for use in adolescents.

One study (NCT02725593) assessed the efficacy of dapagliflozin, an SGLT2 inhibitor, vs. placebo in reducing mean HbA1c in 72 adolescents and young adults. To our knowledge, trial results have not been published in a peer-reviewed journal. However, data submitted to clinicaltrials.gov indicate that there was no significant difference in the change in HbA1c or fasting plasma glucose from baseline to week 24 between dapagliflozin and placebo. Another study assessed the pharmacokinetic and pharmacodynamic profiles of various doses of empagliflozin, another SGLT2 inhibitor, in 27 adolescents (mean age 14.1 years) and found a mean decrease in fasting plasma glucose, a dose-dependent increase in urinary glucose excretion, and no serious adverse events [83].

Phase 3 trials for dapagliflozin (NCT03199053) and empagliflozin (NCT03429543) in children and adolescents are ongoing and estimated to be completed in 2023. Both trials are following a similar design, randomizing an estimated 243 and 186 participants, respectively, 1:1:1 to placebo, an SGLT2 inhibitor (dapagliflozin or empagliflozin), or a DPP4 inhibitor (saxagliptin or linagliptin). Both trials will assess efficacy in change in HbA1c from baseline at week 26 and will continue to follow participants to week 52. Several other SGLT2 inhibitors are currently in various phases of clinical trials in adults and adolescents with T2D (Table 3).

#### SGLT2 inhibitors for T1D

At present, SGLT2 inhibitors are only approved for use in adults with T2D but their efficacy in treating T1D is currently being investigated [88]. Only one trial is currently investigating the use of an SGLT2 inhibitor in adolescents (NCT04333823) and, notably, it is the only trial of a new diabetes agent in adolescents with a kidney-related primary outcome. The Adolescent Type 1 Diabetes Treatment with SGLT2i for hyperglycEMia and hyPerfiltration Trial (ATTEMPT) plans to recruit 100 adolescents (age 12–18 years) with T1D for at least 1 year; participants will be randomized to receive dapagliflozin or placebo once daily for 16 weeks. The primary outcome measure of the trial is change in measured GFR from baseline to week 16, with the goal of understanding the physiological effects of SGLT-2 inhibition on early-onset diabetes complications in youth. This study is estimated to be completed in 2023.

### Surgical intervention

Bariatric surgery has profound, rapid, and long-lasting effects on improving weight loss, cardiovascular risk factors, and glycemic control in adults with T2D, leading to remission of diabetes in over half of all patients after 2 years [92]; surgery has also been shown to reduce the incidence of albuminuria and slow DKD progression [93].

Bariatric surgery is becoming a more accepted treatment option for morbidly obese youth. The Teen-Longitudinal Assessment of Bariatric Surgery (Teen-LABS) study enrolled 242 adolescents undergoing bariatric surgery into a prospective observational study [94]. The most common surgical procedure was Roux-en-Y gastric bypass (66.5%), followed by sleeve gastrectomy (27.7%) and adjustable gastric band (5.8%). Teen-LABS demonstrated that bariatric surgery led to a significant decrease in BMI within 6 months, and that this weight loss was maintained at 3 years. This was associated with significant improvements in eGFR and albuminuria at 3 years [94]. A secondary analysis found that adolescents were 27% more likely than adults to achieve remission of T2D, and 51% more likely than adults to achieve remission of hypertension 5 years after bariatric surgery [95].

Further evidence suggests that bariatric surgery has greater advantages over current standard-of-care lifestyle and pharmacologic options for adolescents with T2D. A secondary analysis compared surgical vs. medical management of T2D in adolescents by matching 30 Teen-LAB participants at the time of surgery with 63 adolescents from the TODAY study. Participants were matched for age, sex, race/ethnicity, and baseline BMI, irrespective of the TODAY treatment group. After 5 years of follow-up, Teen-LABS participants had lower BMI, mean HbA1c, mean triglycerides, and improved insulin sensitivity, whereas TODAY participants had higher BMI, HbA1c, and triglycerides, and worsened insulin sensitivity [96]. Medical management of T2D in adolescents was also associated with poorer kidney outcomes. After 5 years of follow-up, TODAY participants had 27-fold greater odds of elevated urinary AER compared with Teen-LABS participants. However, no randomized controlled trials have done a head-to-head comparison of the effectiveness and safety of surgery to non-surgical conventional treatment options, and therefore, the decision to pursue surgical intervention needs to be individualized and tailored to the unique needs of the patient and the available expertise at the treating institution. In addition, surgical interventions in this young population would require a thorough evaluation of their risks vs. benefits over a much longer follow-up. In general, the guidelines used as an indication for metabolic surgery in adolescents include BMI > 35 kg/m^2^ with comorbidities or BMI > 40 kg/m^2^ with or without comorbidities.

### Transition of care

The period of transition from pediatric to adult care is one associated with deterioration in glycemic control; increased occurrence of acute complications; psychosocial, emotional, and behavioral challenges; and the emergence of chronic complications [97, 98]. Moreover, the process of transition from pediatric to adult care is prone to lead to fragmentation in health care delivery, which may adversely impact health care quality, cost, and outcomes [99].

ADA guidelines recommend that providers prepare youth for transition to adult health care in early adolescence and, at the latest, at least 1 year before transition occurs [25, 98]. During this time, both pediatric and adult diabetes care providers should provide support and resources for transitioning youth. Given the variability among youth in gaining independence and managing both the logistical and medical aspects of diabetes care, the formal transfer of care should only occur when it is deemed appropriate by the patient and the provider.

Strategies to better prepare youth for transition include a directed focus on diabetes self-management skills for the emerging adult with a gradual transfer of diabetes care responsibilities to the teen from the parent or guardian, sharing of information about the differences between pediatric and adult providers in their approaches to care, and education regarding health insurance options and how to maintain coverage. Ideally, the patient and future diabetes provider should also receive a written summarization of all factors related to the patient’s diabetes management [98]. For more details, the reader is referred to the ADA Transitions Workgroup report [98]. While currently there are limited evidence-based strategies for optimal transition of care for youth with diabetes, this is an area of great importance and one that is receiving increased attention; we are hopeful that results of clinical trials such as the “Evaluating Innovations in Transition From Pediatric to Adult Care—The Transition Navigator Trial” (NCT03342495) will help provide a framework to allow the provision of more seamless and high-quality care to such youth.

## Conclusions

In summary, diabetes and DKD are increasingly encountered in children and adolescents. At the same time, our understanding of the pathophysiology of the disease has also increased, as has the armamentarium of therapeutic strategies available to us. Most promising are the three novel classes of mediations which have thus far been largely studied in adult patients: GLP1 receptor agonists, DPP4 inhibitors, and SGLT2 inhibitors, as well as dietary approaches to reverse diabetes and its associated complications (e.g., VLEDs). Many of these interventions have the potential to reduce end-organ damage. Considering the increasing prevalence of diabetes in children and young adults and the more aggressive phenotype of diabetes in this highly vulnerable population, it is time to refocus our efforts and resources on this area by conducting larger and more rigorous clinical trials of these novel therapeutic agents in children and youth with diabetes.

## Key summary points

1. The incidence of diabetes, particularly type 2 diabetes, and its complications, are on the rise in children and adolescents, disproportionately affecting racial-ethnic minorities.
2. The cornerstone in the prevention of diabetic kidney disease is optimal glycemic control, along with screening for and management of hypertension and albuminuria.
3. GLP1 receptor agonists, in conjunction with metformin, have been shown to have a beneficial effect in reducing the incidence of adverse kidney outcomes, and are now approved for use in older children with type 2 diabetes.
4. While many new therapies have been studied and approved for use in adults with diabetes and diabetic kidney disease, insufficient progress has been made in performing clinical trials in children and young adults.

## Multiple choice questions

1. Which of the following statements accurately describes the relationship between albuminuria and progression of DKD?
  - Progression of DKD is universally preceded by the development of albuminuria.
  - Most pediatric patients with microalbuminuria progress to macroalbuminuria.
  - Macroalbuminuria is associated with a higher risk of progressive CKD.
  - Macroalbuminuria invariably progresses to advanced CKD.
2. Which of the following reflects the cumulative incidence of CKD stage 5 in pediatric patients with T1D, 30 years after the onset of disease?
  -  < 10%
  - 10–25%
  - 25–30%
  -  > 30%
3. When should screening for albuminuria start in children and adolescents with T2D?
  - At the time of diagnosis
  - After 1 year of diagnosis
  - After 5 years of diagnosis
  - When diabetic retinopathy is first diagnosed
4. Which of the following statements regarding the development of AKI during DKA is true?
  - AKI is a rare occurrence in children with DKA.
  - The most common cause of AKI in DKA is nephrotoxic ATN.
  - Adults with DKA who develop AKI have a more rapid progression of DKD.
  - Younger age is associated with a higher risk for children with DKA to develop AKI.
5. Which of the following is the most important risk factor associated with the development of DKD in patients with diabetes?
  - Hypertension
  - Insulin resistance
  - Serum uric acid
  - Hyperglycemia

## References

[1] Divers J, Mayer-Davis E, Lawrence J, Isom S (2020). Trends in Incidence of Type 1 and Type 2 Diabetes Among Youths - Selected Counties and Indian Reservations, United States, 2002–2015. MMWR Morb Mortal Wkly Rep.

[2] Kahkoska A, Isom S, Divers J, Mayer-Davis E (2018). The early natural history of albuminuria in young adults with youth-onset type 1 and type 2 diabetes. J Diabetes Complications.

[3] The Restoring Insulin Secretion (RISE) Consortium (2018). Impact of Insulin and Metformin Versus Metformin Alone on β-Cell Function in Youth With Impaired Glucose Tolerance or Recently Diagnosed Type 2 Diabetes. Diabetes Care.

[4] Hannon T, Edelstein S, Arslanian S, Caprio S (2020). Withdrawal of medications leads to worsening of OGTT parameters in youth with impaired glucose tolerance or recently-diagnosed type 2 diabetes. Pediatr Diabetes.

[5] Nadeau K, Anderson B, Berg E, Chiang J (2016). Youth-Onset Type 2 Diabetes Consensus Report: Current Status, Challenges, and Priorities. Diabetes Care.

[6] Bjornstad P, Nehus E, El Ghormli L, Bacha F (2018). Insulin Sensitivity and Diabetic Kidney Disease in Children and Adolescents With Type 2 Diabetes: An Observational Analysis of Data From the TODAY Clinical Trial. Am J Kidney Dis.

[7] Li L, Jick S, Breitenstein S, Michel A (2016). Prevalence of Diabetes and Diabetic Nephropathy in a Large U.S. Commercially Insured Pediatric Population, 2002–2013. Diabetes Care.

[8] Dart A, Sellers E, Martens P, Rigatto C (2012). High burden of kidney disease in youth-onset type 2 diabetes. Diabetes Care.

[9] Orchard TJ, Secrest AM, Miller RG, Costacou T (2010). In the absence of renal disease, 20 year mortality risk in type 1 diabetes is comparable to that of the general population: a report from the Pittsburgh Epidemiology of Diabetes Complications Study. Diabetologia.

[10] Afkarian M, Sachs M, Kestenbaum B, Hirsch I (2013). Kidney disease and increased mortality risk in type 2 diabetes. J Am Soc Nephrol.

[11] Osterby R (1972). Morphometric studies of the peripheral glomerular basement membrane in early juvenile diabetes. I. Development of initial basement membrane thickening. Diabetologia.

[12] Sasson A, Cherney D (2012). Renal hyperfiltration related to diabetes mellitus and obesity in human disease. World J Diabetes.

[13] Tonneijck L, Muskiet M, Smits M, van Bommel E (2017). Glomerular Hyperfiltration in Diabetes: Mechanisms, Clinical Significance, and Treatment. J Am Soc Nephrol.

[14] Ruggenenti P, Porrini E, Gaspari F, Motterlini N (2012). Glomerular hyperfiltration and renal disease progression in type 2 diabetes. Diabetes Care.

[15] Steinke J, Sinaiko A, Kramer M, Suissa S (2005). The early natural history of nephropathy in Type 1 Diabetes: III. Predictors of 5-year urinary albumin excretion rate patterns in initially normoalbuminuric patients. Diabetes.

[16] Murussi M, Gross J, Silveiro S (2006). Glomerular filtration rate changes in normoalbuminuric and microalbuminuric Type 2 diabetic patients and normal individuals A 10-year follow-up. J Diabetes Complications.

[17] Bjornstad P, Cherney D, Snell-Bergeon J, Pyle L (2015). Rapid GFR decline is associated with renal hyperfiltration and impaired GFR in adults with Type 1 diabetes. Nephrol Dial Transplant.

[18] Moriya T, Tanaka S, Sone H, Ishibashi S (2017). Patients with type 2 diabetes having higher glomerular filtration rate showed rapid renal function decline followed by impaired glomerular filtration rate: Japan Diabetes Complications Study. J Diabetes Complications.

[19] Molitch ME, Gao X, Bebu I, de Boer IH (2019). Early Glomerular Hyperfiltration and Long-Term Kidney Outcomes in Type 1 Diabetes: The DCCT/EDIC Experience. Clin J Am Soc Nephrol.

[20] Amin R, Widmer B, Prevost A, Schwarze P (2008). Risk of microalbuminuria and progression to macroalbuminuria in a cohort with childhood onset type 1 diabetes: prospective observational study. BMJ.

[21] Eppens M, Craig M, Cusumano J, Hing S (2006). Prevalence of diabetes complications in adolescents with type 2 compared with type 1 diabetes. Diabetes Care.

[22] de Boer IH, Rue TC, Cleary PA, Lachin JM (2011). Long-term renal outcomes of patients with type 1 diabetes mellitus and microalbuminuria: an analysis of the Diabetes Control and Complications Trial/Epidemiology of Diabetes Interventions and Complications cohort. Arch Intern Med.

[23] de Boer I, Afkarian M, Rue T, Cleary P (2014). Renal outcomes in patients with type 1 diabetes and macroalbuminuria. J Am Soc Nephrol.

[24] Molitch M, Steffes M, Sun W, Rutledge B (2010). Development and progression of renal insufficiency with and without albuminuria in adults with type 1 diabetes in the diabetes control and complications trial and the epidemiology of diabetes interventions and complications study. Diabetes Care.

[25] American Diabetes Association (2021). 13. Children and Adolescents: *Standards of Medical Care in Diabetes-2021*. Diabetes Care.

[26] Donaghue K, Marcovecchio M, Wadwa R, Chew E (2018). ISPAD Clinical Practice Consensus Guidelines 2018: Microvascular and macrovascular complications in children and adolescents. Pediatr Diabetes.

[27] Schwartz G, Muñoz A, Schneider M, Mak R (2009). New equations to estimate GFR in children with CKD. J Am Soc Nephrol.

[28] Boettcher C, Utsch B, Galler A, Grasemann C (2020). Estimated Glomerular Filtration Rates Calculated by New and Old Equations in Children and Adolescents With Type 1 Diabetes-What to Do With the Results?. Front Endocrinol (Lausanne).

[29] Winnicki E, McCulloch C, Mitsnefes M, Furth S (2018). Use of the Kidney Failure Risk Equation to Determine the Risk of Progression to End-stage Renal Disease in Children With Chronic Kidney Disease. JAMA Pediatr.

[30] Marcovecchio M, Tossavainen P, Dunger D (2009). Status and rationale of renoprotection studies in adolescents with type 1 diabetes. Pediatr Diabetes.

[31] Gallego PH, Shephard N, Bulsara MK, van Bockxmeer FM (2008). Angiotensinogen gene T235 variant: a marker for the development of persistent microalbuminuria in children and adolescents with type 1 diabetes mellitus. J Diabetes Complicat.

[32] Marcovecchio ML, Dalton RN, Turner C, Prevost AT (2010). Symmetric dimethylarginine, an endogenous marker of glomerular filtration rate, and the risk for microalbuminuria in young people with type 1 diabetes. Arch Dis Child.

[33] Seyfarth J, Herebian D, Reinauer C, Baechle C (2020). Evaluation of lipoprotein-associated phospholipase A2 as a marker for renal microvasculopathy in adolescents with Type 1 diabetes. Diabet Med.

[34] Beisswenger P, Howell S, Russell G, Miller M (2014). Detection of diabetic nephropathy from advanced glycation endproducts (AGEs) differs in plasma and urine, and is dependent on the method of preparation. Amino Acids.

[35] Wheelock K, Cai J, Looker H, Merchant M (2017). Plasma bradykinin and early diabetic nephropathy lesions in type 1 diabetes mellitus. PLoS One.

[36] Gohda T, Niewczas M, Ficociello L, Walker W (2012). Circulating TNF receptors 1 and 2 predict stage 3 CKD in type 1 diabetes. J Am Soc Nephrol.

[37] Sabbisetti V, Waikar S, Antoine D, Smiles A (2014). Blood kidney injury molecule-1 is a biomarker of acute and chronic kidney injury and predicts progression to ESRD in type I diabetes. J Am Soc Nephrol.

[38] Hsu C, Xie D, Waikar S, Bonventre J (2017). Urine biomarkers of tubular injury do not improve on the clinical model predicting chronic kidney disease progression. Kidney Int.

[39] Sanyoura M, Philipson L, Naylor R (2018). Monogenic Diabetes in Children and Adolescents: Recognition and Treatment Options. Curr Diab Rep.

[40] Hursh B, Ronsley R, Islam N, Mammen C (2017). Acute Kidney Injury in Children With Type 1 Diabetes Hospitalized for Diabetic Ketoacidosis. JAMA Pediatr.

[41] Myers S, Glaser N, Trainor J, Nigrovic L (2020). Frequency and Risk Factors of Acute Kidney Injury During Diabetic Ketoacidosis in Children and Association With Neurocognitive Outcomes. JAMA Netw Open.

[42] Orban J, Maiziere E, Ghaddab A, Van Obberghen E (2014). Incidence and characteristics of acute kidney injury in severe diabetic ketoacidosis. PLoS One.

[43] Thakar C, Christianson A, Himmelfarb J, Leonard A (2011). Acute kidney injury episodes and chronic kidney disease risk in diabetes mellitus. Clin J Am Soc Nephrol.

[44] Chen J, Zeng H, Ouyang X, Zhu M (2020). The incidence, risk factors, and long-term outcomes of acute kidney injury in hospitalized diabetic ketoacidosis patients. BMC Nephrol.

[45] Yu S, Bonventre J (2018). Acute Kidney Injury and Progression of Diabetic Kidney Disease. Adv Chronic Kidney Dis.

[46] (1994) Effect of intensive diabetes treatment on the development and progression of long-term complications in adolescents with insulin-dependent diabetes mellitus: Diabetes Control and Complications Trial. Diabetes Control and Complications Trial Research Group. J Pediatr 125:177–188 10.1016/s0022-3476(94)70190-3 8040759

[47] Selvin E, Steffes M, Zhu H, Matsushita K (2010). Glycated hemoglobin, diabetes, and cardiovascular risk in nondiabetic adults. N Engl J Med.

[48] Selvin E, Coresh J, Golden S, Boland L (2005). Glycemic control, atherosclerosis, and risk factors for cardiovascular disease in individuals with diabetes: the atherosclerosis risk in communities study. Diabetes Care.

[49] Nathan D, Genuth S, Lachin J, Cleary P (1993). The effect of intensive treatment of diabetes on the development and progression of long-term complications in insulin-dependent diabetes mellitus. N Engl J Med.

[50] Agrawal L, Azad N, Bahn G, Reaven P (2019). Intensive Glycemic Control Improves Long-term Renal Outcomes in Type 2 Diabetes in the Veterans Affairs Diabetes Trial (VADT). Diabetes Care.

[51] Wong M, Perkovic V, Chalmers J, Woodward M (2016). Long-term Benefits of Intensive Glucose Control for Preventing End-Stage Kidney Disease: ADVANCE-ON. Diabetes Care.

[52] Gerstein HC, Miller ME, Genuth S, Ismail-Beigi F (2011). Long-term effects of intensive glucose lowering on cardiovascular outcomes. N Engl J Med.

[53] Helliwell R, Warnes H, Kietsiriroje N, Campbell M (2021). Body mass index, estimated glucose disposal rate and vascular complications in type 1 diabetes: beyond glycated haemoglobin. Diabet Med.

[54] Melsom T, Mathisen U, Ingebretsen O, Jenssen T (2011). Impaired fasting glucose is associated with renal hyperfiltration in the general population. Diabetes Care.

[55] Nadeau K, Regensteiner J, Bauer T, Brown M (2010). Insulin resistance in adolescents with type 1 diabetes and its relationship to cardiovascular function. J Clin Endocrinol Metab.

[56] TODAY Study Group (2013). Rapid rise in hypertension and nephropathy in youth with type 2 diabetes: the TODAY clinical trial. Diabetes Care.

[57] The Restoring Insulin Secretion (RISE) Consortium (2018). Metabolic Contrasts Between Youth and Adults With Impaired Glucose Tolerance or Recently Diagnosed Type 2 Diabetes: I. Observations Using the Hyperglycemic Clamp. Diabetes Care.

[58] Soergel M, Schaefer F (2002). Effect of hypertension on the progression of chronic renal failure in children. Am J Hypertens.

[59] Flynn J, Kaelber D, Baker-Smith C, Blowey D (2017). Clinical Practice Guideline for Screening and Management of High Blood Pressure in Children and Adolescents. Pediatrics.

[60] Bjornstad P, Laffel L, Lynch J, El Ghormli L (2019). Elevated Serum Uric Acid Is Associated With Greater Risk for Hypertension and Diabetic Kidney Diseases in Obese Adolescents With Type 2 Diabetes: An Observational Analysis From the Treatment Options for Type 2 Diabetes in Adolescents and Youth (TODAY) Study. Diabetes Care.

[61] Goicoechea M, Garcia de Vinuesa S, Verdalles U, Verde E (2015). Allopurinol and progression of CKD and cardiovascular events: long-term follow-up of a randomized clinical trial. Am J Kidney Dis.

[62] Doria A, Galecki A, Spino C, Pop-Busui R (2020). Serum Urate Lowering with Allopurinol and Kidney Function in Type 1 Diabetes. N Engl J Med.

[63] Kimura K, Hosoya T, Uchida S, Inaba M (2018). Febuxostat Therapy for Patients With Stage 3 CKD and Asymptomatic Hyperuricemia: A Randomized Trial. Am J Kidney Dis.

[64] Sauder K, Stafford J, The N, Mayer-Davis E (2020). Dietary strategies to manage diabetes and glycemic control in youth and young adults with youth-onset type 1 and type 2 diabetes: the SEARCH for diabetes in youth study. Pediatr Diabetes.

[65] Nip A, Reboussin B, Dabelea D, Bellatorre A (2019). Disordered Eating Behaviors in Youth and Young Adults With Type 1 or Type 2 Diabetes Receiving Insulin Therapy: the SEARCH for Diabetes in Youth Study. Diabetes Care.

[66] Zeitler P, Hirst K, Pyle L, Linder B (2012). A clinical trial to maintain glycemic control in youth with type 2 diabetes. N Engl J Med.

[67] Lean M, Leslie W, Barnes A, Brosnahan N (2019). Durability of a primary care-led weight-management intervention for remission of type 2 diabetes: 2-year results of the DiRECT open-label, cluster-randomised trial. Lancet Diabetes Endocrinol.

[68] Andela S, Burrows T, Baur L, Coyle D (2019). Efficacy of very low-energy diet programs for weight loss: a systematic review with meta-analysis of intervention studies in children and adolescents with obesity. Obes Rev.

[69] Williams D, Nawaz A, Evans M (2020). Renal Outcomes in Type 2 Diabetes: a Review of Cardiovascular and Renal Outcome Trials. Diabetes Ther.

[70] Cefalu W, Kaul S, Gerstein H, Holman R (2018). Cardiovascular Outcomes Trials in Type 2 Diabetes: where Do We Go From Here? Reflections From a Diabetes Care Editors’ Expert Forum. Diabetes Care.

[71] American Diabetes Association (2021). 9. Pharmacologic Approaches to Glycemic Treatment: standards of Medical Care in Diabetes-2021. Diabetes Care.

[72] Arslanian S, Bacha F, Grey M, Marcus M (2018). Evaluation and Management of Youth-Onset Type 2 Diabetes: a Position Statement by the American Diabetes Association. Diabetes Care.

[73] de Boer I, Caramori M, Chan J, Heerspink H (2020). KDIGO 2020 Clinical Practice Guideline for Diabetes Management in Chronic Kidney Disease. Kidney Int.

[74] Raman V, Mason K, Rodriguez L, Hassan K (2010). The role of adjunctive exenatide therapy in pediatric type 1 diabetes. Diabetes Care.

[75] Griffin K, Thompson P, Gottschalk M, Kyllo J (2014). Combination therapy with sitagliptin and lansoprazole in patients with recent-onset type 1 diabetes (REPAIR-T1D): 12-month results of a multicentre, randomised, placebo-controlled, phase 2 trial. Lancet Diabetes Endocrinol.

[76] Tamborlane W, Barrientos-Pérez M, Fainberg U, Frimer-Larsen H (2019). Liraglutide in Children and Adolescents with Type 2 Diabetes. N Engl J Med.

[77] Klein DJ, Battelino T, Chatterjee DJ, Jacobsen LV (2014). Liraglutide’s safety, tolerability, pharmacokinetics, and pharmacodynamics in pediatric type 2 diabetes: a randomized, double-blind, placebo-controlled trial. Diabetes Technol Ther.

[78] Petri K, Jacobsen L, Klein D (2015). Comparable liraglutide pharmacokinetics in pediatric and adult populations with type 2 diabetes: a population pharmacokinetic analysis. Clin Pharmacokinet.

[79] Malloy J, Capparelli E, Gottschalk M, Guan X (2009). Pharmacology and tolerability of a single dose of exenatide in adolescent patients with type 2 diabetes mellitus being treated with metformin: a randomized, placebo-controlled, single-blind, dose-escalation, crossover study. Clin Ther.

[80] Tamborlane W, Laffel L, Weill J, Gordat M (2018). Randomized, double-blind, placebo-controlled dose-finding study of the dipeptidyl peptidase-4 inhibitor linagliptin in pediatric patients with type 2 diabetes. Pediatr Diabetes.

[81] Fraser I, Neufeld N, Fox L, Kipnes M (2019). A randomized clinical trial to evaluate the single-dose pharmacokinetics, pharmacodynamics, and safety of sitagliptin in pediatric patients with type 2 diabetes. Pediatr Diabetes.

[82] Parkinson J, Tang W, Johansson C, Boulton D (2016). Comparison of the exposure-response relationship of dapagliflozin in adult and paediatric patients with type 2 diabetes mellitus. Diabetes Obes Metab.

[83] Laffel L, Tamborlane W, Yver A, Simons G (2018). Pharmacokinetic and pharmacodynamic profile of the sodium-glucose co-transporter-2 inhibitor empagliflozin in young people with Type 2 diabetes: a randomized trial. Diabet Med.

[84] Bjornstad P, Laffel L, Tamborlane W, Simons G (2018). Acute Effect of Empagliflozin on Fractional Excretion of Sodium and eGFR in Youth With Type 2 Diabetes. Diabetes Care.

[85] Kristensen S, Rørth R, Jhund P, Docherty K (2019). Cardiovascular, mortality, and kidney outcomes with GLP-1 receptor agonists in patients with type 2 diabetes: a systematic review and meta-analysis of cardiovascular outcome trials. Lancet Diabetes Endocrinol.

[86] Mikov M, Pavlović N, Stanimirov B, Đanić M (2020). DPP-4 Inhibitors: Renoprotective Potential and Pharmacokinetics in Type 2 Diabetes Mellitus Patients with Renal Impairment. Eur J Drug Metab Pharmacokinet.

[87] Mann J, Muskiet M (2021). Incretin-based drugs and the kidney in type 2 diabetes: choosing between DPP-4 inhibitors and GLP-1 receptor agonists. Kidney Int.

[88] Avgerinos I, Manolopoulos A, Michailidis T, Kitsios K (2021). Comparative efficacy and safety of glucose-lowering drugs as adjunctive therapy for adults with type 1 diabetes: A systematic review and network meta-analysis. Diabetes Obes Metab.

[89] Williams D, Nawaz A, Evans M (2021). Sodium-Glucose Co-Transporter 2 (SGLT2) Inhibitors: Are They All the Same? A Narrative Review of Cardiovascular Outcome Trials. Diabetes Ther.

[90] Barbarawi M, Al-Abdouh A, Barbarawi O, Lakshman H (2021). SGLT2 inhibitors and cardiovascular and renal outcomes: a meta-analysis and trial sequential analysis. Heart Fail Rev.

[91] Palmer S, Tendal B, Mustafa R, Vandvik P (2021). Sodium-glucose cotransporter protein-2 (SGLT-2) inhibitors and glucagon-like peptide-1 (GLP-1) receptor agonists for type 2 diabetes: systematic review and network meta-analysis of randomised controlled trials. BMJ.

[92] Khorgami Z, Shoar S, Saber A, Howard C (2019). Outcomes of Bariatric Surgery Versus Medical Management for Type 2 Diabetes Mellitus: a Meta-Analysis of Randomized Controlled Trials. Obes Surg.

[93] Martin W, White J, López-Hernández F, Docherty N (2020). Metabolic Surgery to Treat Obesity in Diabetic Kidney Disease, Chronic Kidney Disease, and End-Stage Kidney Disease; What Are the Unanswered Questions?. Front Endocrinol (Lausanne).

[94] Nehus E, Khoury J, Inge T, Xiao N (2017). Kidney outcomes three years after bariatric surgery in severely obese adolescents. Kidney Int.

[95] Inge T, Courcoulas A, Jenkins T, Michalsky M (2019). Five-Year Outcomes of Gastric Bypass in Adolescents as Compared with Adults. N Engl J Med.

[96] Bjornstad P, Hughan K, Kelsey M, Shah A (2020). Effect of Surgical Versus Medical Therapy on Diabetic Kidney Disease Over 5 Years in Severely Obese Adolescents With Type 2 Diabetes. Diabetes Care.

[97] Bryden K, Peveler R, Stein A, Neil A (2001). Clinical and psychological course of diabetes from adolescence to young adulthood: a longitudinal cohort study. Diabetes Care.

[98] Peters A, Laffel L; American Diabetes Association Transitions Working Group (2011). Diabetes care for emerging adults: recommendations for transition from pediatric to adult diabetes care systems: a position statement of the American Diabetes Association, with representation by the American College of Osteopathic Family Physicians, the American Academy of Pediatrics, the American Association of Clinical Endocrinologists, the American Osteopathic Association, the Centers for Disease Control and Prevention, Children with Diabetes, The Endocrine Society, the International Society for Pediatric and Adolescent Diabetes, Juvenile Diabetes Research Foundation International, the National Diabetes Education Program, and the Pediatric Endocrine Society (formerly Lawson Wilkins Pediatric Endocrine Society). Diabetes Care.

[99] Mays J, Jackson K, Derby T, Behrens J (2016). An Evaluation of Recurrent Diabetic Ketoacidosis, Fragmentation of Care, and Mortality Across Chicago, Illinois. Diabetes Care.

